# Impact of intravenous exenatide infusion for perioperative blood glucose control on myocardial ischemia-reperfusion injuries after coronary artery bypass graft surgery: sub study of the phase II/III ExSTRESS randomized trial

**DOI:** 10.1186/s12933-018-0784-y

**Published:** 2018-11-01

**Authors:** Guillaume Besch, Andrea Perrotti, Lucie Salomon du Mont, Marc Puyraveau, Xavier Ben-Said, Maude Baltres, Benoit Barrucand, Guillaume Flicoteaux, Lucie Vettoretti, Emmanuel Samain, Sidney Chocron, Sebastien Pili-Floury

**Affiliations:** 1 Department of Anesthesiology and Intensive Care Medicine, University Hospital of Besancon, and, EA3920 and SFR-FED 4234 INSERM, University of Franche-Comte, 3 bvd Alexander Fleming, 25000 Besançon, France; 2 Department of Cardiothoracic Surgery, University of Franche-Comte, 3 bvd Alexander Fleming, 25000 Besançon, France; 3 Department of Vascular Surgery, University Hospital of Besancon, and, EA3920, University of Franche-Comte, 3 bvd Alexander Fleming, 25000 Besançon, France; 4 Clinical Methodology Center, University Hospital of Besancon, University of Franche-Comte, 3 bvd Alexander Fleming, 25000 Besançon, France; 5 Department of Cardiothoracic Surgery, University Hospital of Besancon, and, EA3920, University of Franche-Comte, 3 bvd Alexander Fleming, 25000 Besançon, France

**Keywords:** Coronary artery bypass, Exenatide, Incretins, Glucagon-like peptide 1, Myocardial reperfusion injury, Cardioprotective effects

## Abstract

**Background:**

The aim of the study was to investigate whether intravenous (iv) infusion of exenatide, a synthetic GLP-1 receptor agonist, could provide a protective effect against myocardial ischemia-reperfusion injury after coronary artery bypass graft (CABG) surgery.

**Methods:**

A sub study analysis of patients > 18 years admitted for elective CABG and included in the ExSTRESS trial was conducted. Patients were randomized to receive either iv exenatide (1-h bolus of 0.05 µg min^−1^ followed by a constant infusion of 0.025 µg min^−1^) (exenatide group) or iv insulin therapy (control group) for blood glucose control (target range 100–139 mg dl^−1^) during the first 48 h after surgical incision. All serum levels of troponin I measured during routine care in the Cardiac Surgery ICU were recorded. The primary outcome was the highest value of plasma concentration of troponin I measured between 12 and 24 h after ICU admission. The proportion of patients presenting an echocardiographic left ventricular ejection fraction (LVEF) > 50% at the follow-up consultation was compared between the two groups.

**Results:**

Finally, 43 and 49 patients were analyzed in the control and exenatide groups, respectively {age: 69 [61–76] versus 71 [63–75] years; baseline LVEF < 50%: 6 (14%) versus 16 (32%) patients; on-pump surgery: 29 (67%) versus 33 (67%) patients}. The primary outcome did not significantly differ between the two groups (3.34 [1.06–6.19] µg l^−1^ versus 2.64 [1.29–3.85] µg l^−1^ in the control and exenatide groups, respectively; mean difference (MD) [95% confidence interval (95% CI)] 0.16 [− 0.25; 0.57], p = 0.54). The highest troponin value measured during the first 72 h in the ICU was 6.34 [1.36–10.90] versus 5.04 [2.39–7.18] µg l^−1^, in the control and exenatide groups respectively (MD [95% CI] 0.20 [− 0.22; 0.61], p = 0.39). At the follow-up consultation, 5 (12%) versus 8 (16%) patients presented a LVEF < 50% in the control and in the exenatide groups respectively (relative risk [95% CI] 0.68 [0.16; 2.59], p = 0.56).

**Conclusions:**

Postoperative iv exenatide did not provide any additional cardioprotective effect compared to iv insulin in low-risk patients undergoing scheduled CABG surgery.

*Trial registration* ClinicalTrials.gov Identifier NCT01969149, date of registration: January 7th, 2015; EudraCT No. 2009-009254-25 A, date of registration: January 6th, 2009

## Background

Temporary interruption of coronary artery blood flow during coronary artery bypass graft (CABG) surgery can cause myocardial lesions during both the ischemic and the reperfusion phases. Brief episodes of ischemia before (pre-conditioning) or after (post-conditioning) interruption of the coronary blood flow is believed to activate protective intracellular mechanisms, thus preventing the induction of apoptosis and subsequent death of cardiomyocytes [1]. Several pharmaceutical products, including the glucagon-like peptide 1 (GLP-1) and its agonists, are known to trigger pre- and post-conditioning [2]. The GLP-1 has been reported to exert cardioprotective effects in several animal studies of myocardial ischemia-reperfusion through the activation of the G protein—coupled GLP-1 receptor present in myocytes [3–9]. A reduction in myocardial infarct size was reported after administration of GLP-1 or its metabolites during the ischemia and/or reperfusion phases [3–10]. This cardioprotective effect of GLP-1 could explain the lesser degree of alteration in left ventricular function, and the reduced need for inotropic agents reported in patients undergoing CABG, and in animal models of myocardial ischemia-reperfusion injury [3–9, 11, 12]. It has also been suggested that GLP-1 may have proper inotropic effects [11–14], and could contribute to improving quality of life [14]. However, GLP-1 is not available in daily routine practice, and its use is strictly limited to the research context.

Exenatide is a synthetic GLP-1 receptor agonist. Intravenous (iv) administration of exenatide during the first 6 h after myocardial infarction has been shown to reduce infarct size [15–17], through a cardioprotective mechanism independent of the improvement of glycemic control [18], implying the activation of G protein—coupled GLP-1 receptor present in myocytes [19]. To date, cardioprotective effects of exenatide have been yet to be investigated in other patient populations.

The hypothesis of this study was that iv exenatide could provide pharmacologic postconditioning against myocardial ischemia-reperfusion injury, and thus improve left ventricular function and quality of life after CABG surgery. The aim of this study was to investigate whether iv perioperative administration of exenatide would reduce cardiac enzymes release resulting from ischemia/reperfusion injury in patients undergoing scheduled CABG surgery.

## Methods

### Study design

This is a sub study of the single-center, randomized, open-label, phase III/IV ExSTRESS trial, performed from January to December 2015. The aim of the ExSTRESS trial was to investigate whether perioperative iv administration of exenatide improved glycemic control in patients undergoing scheduled CABG surgery, and the main findings have previously been reported elsewhere [20]. The study was approved by the local Ethics Committee (CPP Est-II, Centre Hospitalier Universitaire de Besançon under the number 09/503/429, President: Prof. Jean-Pierre Kantelip) on November 25, 2010, and by the national agency for the safety of medical products (Agence Nationale de Sécurité du Médicaments et des produits de santé, ANSM) on July 11, 2013. The protocol was registered with the European Union Drug Regulating Authorities Clinical Trials (EudraCT No. 2009-009254-25 A) on January 6, 2009, and was published on ClinicalTrials.gov under the identifier NCT01969149 (principal investigator: Guillaume Besch, M.D., Ph.D) on January 7, 2015, prior to patient enrollment. Written informed consent was obtained from all subjects. This manuscript adheres to the applicable Consolidated Standards of Reporting Trials (CONSORT) guidelines.

### Population of the study

All patients admitted for scheduled CABG surgery were eligible. All eligible patients were informed about the study (oral and written information) at the preoperative anesthesiology consultation at least 2 days before surgery. Patients who provided written informed consent were included on the day before surgery. The exclusion criteria were: age < 18 years; legal incapacity to consent; pregnant or lactating women; emergency surgery; patients with valve replacement and/or thoracic aortic surgery performed during the same procedure; type 1 diabetes or type 2 diabetes treated by insulin; preoperative fasting glycemia > 300 mg dl^−1^; preoperative glycated hemoglobin > 8%; creatinine clearance < 60 ml min^−1^ as assessed by the Modification of Diet in Renal Disease formula [21]; American Society of Anesthesiologists (ASA) physical status > III; diabetic ketoacidosis; hyperosmolar hyperglycemic state; a history of chronic pancreatitis or pancreatectomy; presence of a contraindication to exenatide (Astra Zeneca, Courbevoie, France), insulin lispro (Lilly, France) or human albumin (LFB Biomedicaments, France). Patients were also excluded if they converted to unplanned valve replacement and/or thoracic aortic surgery during the CABG procedure.

### Anesthetic and blood glucose management

All antidiabetic medications were discontinued the day before surgery and patients were fasting from midnight prior to the procedure. Surgery was performed under general anesthesia left at the discretion of the attending anesthesiologist. Post-operative prophylaxis of nausea and vomiting and antibiotic prophylaxis were prescribed in accordance with the recommendations of the French Society of Anesthesia & Intensive Care Medecine (Société Française d’Anesthésie Réanimation, SFAR). Intraoperative hydration was based on saline infusion. No infusion of glucose solution was given in the operating theatre in the absence of hypoglycemia (glycemia < 75 mg dl^−1^).

Immediately after surgery, all patients were admitted to the Cardiac Surgery Intensive Care Unit (ICU). Sedation was discontinued and extubation performed as early as possible, according to usual criteria. Intravenous infusion of glucose solution at a dose of 4.0–4.5 g h^−1^ was prescribed until oral feeding was resumed. Post-operative analgesia was based on an association of continuous infusion of remifentanil (0.05 µg kg^−1^ min^−1^), continuous infusion of nefopam (120 mg/24 h) and intravenous paracetamol (15 mg kg^−1^/6 h).

In the control group, glycemic control was performed in accordance with the standard intravenous insulin therapy protocol used in the Cardiac Surgery ICU. This protocol has been in force since December 2005 and has previously been described elsewhere [22]. Briefly, insulin therapy was based primarily on the rate of glycemic changes and used three measurements to adjust intravenous insulin infusions: (1) the current blood glucose value; (2) the previous blood glucose value; and (3) the current insulin infusion rate. Glycemia was measured every hour starting from surgical incision. The target blood glucose level was between 100 and 139 mg dl^−1^. Intravenous administration of insulin was initiated as soon as a first value > 139 mg dl^−1^ was observed. Blood glucose monitoring was performed every 3 h after 12 consecutive blood glucose measures between 100 and 139 mg dl^−1^. Hourly blood glucose monitoring was resumed if any of the following occurred: (1) change in insulin infusion rate; (2) change in clinical condition; or (3) initiation or cessation of vasopressor therapy or renal replacement therapy. Interruption of insulin administration and oral or intravenous sugar intake according to a standardized protocol were described in the protocol in case of hypoglycemia.

In the exenatide group, glycemic control was based on intravenous administration of exenatide. To avoid adsorption of exenatide on the infusion tubes, a solution containing 0.2 µg ml^−1^ exenatide and 2 mg ml^−1^ human albumin mixed in a solution of NaCl 0.9% was prepared by the nurse in charge of the patient. Blood glucose controls were performed as for the control group. Continuous intravenous administration of exenatide was initiated as soon as the first blood glucose value > 139 mg dl^−1^ was observed. After a bolus of 15 ml during the first hour, a constant flow rate of 7.5 ml h^−1^ was prescribed. If blood glucose remained > 139 mg dl^−1^ 3 h after initiation of exenatide, then intravenous administration of exenatide was pursued at the same flow rate, and a continuous intravenous infusion of insulin was added, according to the same modalities as for the control group. The flow rates of exenatide and insulin were adapted, and sugar intake either orally or intravenously were prescribed according to a standardized protocol specific to the exenatide group in case of hypoglycemia.

Depending on the patient’s progress, oral food intake was resumed as early as possible. Intravenous administration of exenatide and/or insulin was discontinued as soon as oral food intake was resume, and relayed if necessary by subcutaneous insulin, in accordance with the standardized protocol used in Cardiac Surgery ICU. Intravenous administration of exenatide was discontinued at the latest 48 h after surgical incision and relayed with continuous infusion of insulin in case of persistent hyperglycemia and in the absence of oral food intake. If the patient was transferred to another unit before 48 h, exenatide administration was also discontinued.

### Randomization

Included patients were randomly assigned to one of two groups on the day before surgery, namely the control group or the exenatide group. Randomization was performed using a randomization list generated by computer software and integrated into a web interface (CleanWeb software, Telemedicine technologies, France) by an independent data manager before the start of the study. Randomization was on a 1:1 ratio, in blocks of varying size (2 and 4) and stratified by the presence of diabetes (yes/no). The investigator who obtained the informed consent from the patient entered the data online, inclusion, non-inclusion and stratification criteria were verified, and the patient’s randomized group allocation was then displayed immediately by CleanWeb. The size of the randomization blocks was not known to the investigators. The randomized allocation was noted in the patient’s medical file and was thus available to all the caregivers involved in the patient’s care.

### Data collected and outcomes

Sociodemographic data, comorbidities, ASA physical status and Euroscore value were entered online at the time of inclusion. Glycated hemoglobin was tested routinely in the course of the pre-operative work-up. All data pertaining to the surgical procedure and anesthesia were recorded from the computerized surveillance tab of the anesthesia file.

All blood glucose values measured during the 48 h following surgical incision were recorded. All serum levels of troponin I and brain natriuretic peptide (BNP) measured during routine care in the Cardiac Surgery ICU were also recorded. Briefly, blood troponin I levels were measured at 6 and 12 h after the last anastomosis in off-pump surgery or after cardiopulmonary bypass in on-pump surgery, and then, each morning during the stay in the Cardiac Surgery ICU, unless significant clinical deterioration and/or suspicion of myocardial ischemia [23].

Troponin I values were classed into 4 time intervals, based on admission to the Cardiac Surgery ICU, namely: 0–12 h (troponin_0–12_); 12–24 h (troponin_12–24_); 24–48 h (troponin_24–48_); and 48–72 h (troponin_48–72_). BNP values were categorized into 3 time intervals, according to the time of admission to the Cardiac Surgery ICU, namely: 0–12 h (BNP_0–12_); 12–24 h (BNP_12–24_); and 24–48 h (BNP_24–48_). If two blood tests were performed within a single time interval, the highest troponin or BNP in that time window was recorded. Peak post-operative troponin I was defined as the highest value from among troponin_0–12_, troponin_12–24_, troponin_24–48_, and troponin_48–72_. The area under the curve of troponin I values during the 72 h after admission in the Cardiac Surgery ICU (AUC_troponin_) was calculated by using the trapezoid method, as follows: AUC_troponin_ = ∑ [(X^n^ + X^n−1^) (T^n^ – T^n−1^)]/2, with X^n^, the troponin I value measured at time T^n^, and X^n−1^, the last troponin I value measured at time T^n−1^. We calculated the proportion of patients with BNP_0–12_ ≥ 200 pg ml^−1^, BNP_12–24_ ≥ 200 pg ml^−1^ or BNP_24–48_ ≥ 200 pg ml^−1^.

In order to assess myocardial recovery after ischemia/reperfusion, left ventricular ejection fraction (LVEF) was measured by transthoracic echocardiography at the post-operative follow-up between 3 and 6 months after surgery. The proportion of patients with LVEF < 30%, 30% < LVEF < 50%, and LVEF > 50% at post-operative follow-up was recorded; the categories used are based on those used to calculate the EuroScore value. The administration of catecholamines (noradrenaline, dobutamine, adrenaline) during the stay in the Cardiac Surgery ICU was also noted.

To assess quality of life, the Short Form (SF) 36 questionnaire was completed by patients at the inclusion visit, and then sent to patients by post for completion at 1, 3, 6 and 12 months after surgery. The SF36 questionnaire is validated in the French language [24] and is recommended by the French health authorities (Haute Autorité de Santé, HAS) for the assessment of quality of life in patients with coronary artery disease. The SF36 is a self-report questionnaire that investigates eight dimensions, namely physical function (PF), physical role (PR), bodily pain (BP), global health (GH), vitality (VT), social function (SF), role emotional (RE) and mental health (MH). An overall score between 0 and 100 is calculated by summing the scores for each dimension.

The primary outcome was plasma concentration of troponin I between 12 and 24 h postoperatively (troponin_12–24_). Troponin_12–24_ was considered to be the serum level of troponin at postoperative day 1 depending on whether the surgery was performed on the morning or in the afternoon. Serum level troponin at postoperative day 1 was associated with midterm and long-term outcome after CABG in previous studies [25–27]. Secondary endpoints were: plasma concentration of troponin during the first 72 h in the ICU after surgery; peak post-operative troponin; area under the curve of troponin I values during the 72 h after admission to the Cardiac Surgery ICU (AUC_troponin_); plasma concentration of BNP during the first 48 h in the ICU after surgery; the proportion of patients with BNP_0–12_, BNP_12–24_ and/or BNP_24–48_ ≥ 200 pg ml^−1^; the proportion of patients treated with dobutamine during the ICU stay; the proportion of patients with LVEF < 30%, 30% < LVEF < 50%, and LVEF > 50% at post-operative follow-up; scores on each of the scales of the SF36 at inclusion and at 1, 3, 6 and 12 months.

### Statistical analysis

The Shapiro–Wilk test was used to test the hypothesis of normal distribution for quantitative variables. Quantitative variables are presented as means [standard deviations (SD)] (normally distributed data) or medians (interquartile ranges [IQR] 25–75%) (not normally distributed data) and qualitative variables as number (percentage) unless otherwise stated. Standardized differences were used to assess imbalances between baseline characteristics. Comparisons between the control and exenatide groups were performed using the Chi square or Fisher’s exact test for qualitative variables, and the Student *t* or Mann-Whitney *U* tests for quantitative variables, as appropriate. The repeated measurements of plasma concentrations of troponin and BNP, and of the scores on the different dimensions of the SF36 were compared between groups using repeated measures ANOVA.

All analyses were performed using SAS version 9.4 (SAS Institute Inc., Cary, NC, USA). All statistical tests were 2-sided and a p-value < 0.05 was considered statistically significant.

### Sample size

The present study is a sub study of the ExSTRESS trial. The ExSTRESS study was a randomized phase II/III study. The phase II methodology was performed according to a 2-stage procedure as described by O’Brien and Fleming and has been published elsewhere [20, 28]. The sample size was calculated based on the primary endpoint of the ExSTRESS Study. The inclusion of 55 patients in each group (control and exenatide groups) was planned during phase II. The sample size for the phase III study was calculated based on the data observed in phase II.

The original intent of the present study was to assess whether iv exenatide could improve the quality of life after CABG by allowing a better left ventricular function while reducing myocardial ischemia-reperfusion injuries. A sufficient statistical power to test this hypothesis was anticipated by including all patients included in the phase II and in the phase III of the ExSTRESS trial. However, the ExSTRESS study was prematurely terminated for futility during phase II, in the absence of any clinically relevant benefit yielded by the use of exenatide to control blood glucose in patients undergoing CABG surgery. The sample size included in the phase II of the ExSTRESS study did not provide enough statistical power to compare quality of life between the control and exenatide groups. Thus, the primary hypothesis of the present study was adjusted to investigate the postconditioning effect of iv exenatide after CABG.

The analyses in the current study were performed on the ExSTRESS study population but excluding patients who died in the Cardiac Surgery ICU, patients who had fewer than 3 troponin measures, and patients who received neither insulin nor exenatide. No sample size calculation was performed before data analysis.

## Results

### Study population

**Fig. 1:**
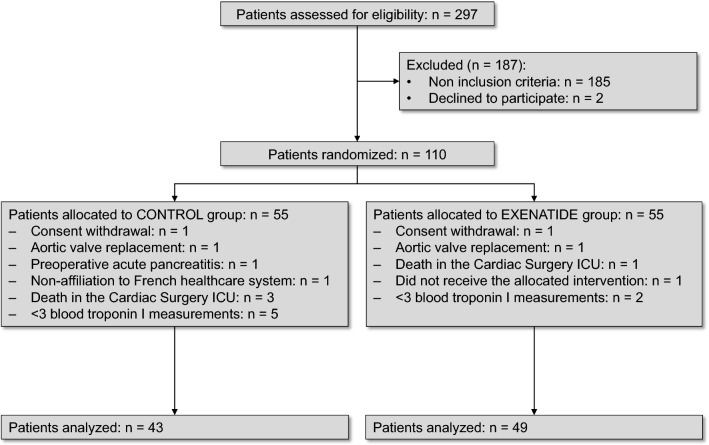
Flow-chart of the study according to the CONSORT statement

In the ExSTRESS study, a total of 297 patients were eligible during the study period. Fifty-five patients were randomized to each group, and 51 and 53 patients were analyzed in the control and exenatide groups respectively. For the present analysis, 8 and 4 patients were excluded from the control and exenatide groups respectively. The reasons for exclusions are detailed in the study flowchart shown in Fig. 1. One patient in the exenatide group received neither insulin nor exenatide. Finally, data from 43 and 49 patients were analyzed from the control and exenatide groups respectively. The sociodemographic data, comorbidities, and procedural characteristics were similar between groups (Table 1). The average time from admission to the Cardiac Surgery ICU and initiation of treatment for glycemic control (with either insulin or intravenous exenatide) was 3 (3) and 3 (4) hours in the control and exenatide groups respectively (p = 0.64). The drugs used for maintenance of anesthesia are presented in Table 1. The anesthesia protocol included administration of sevoflurane in 29 (67%) versus 33 (67%) patients in the control and exenatide groups respectively (p = 0.99). Among the patients of the exenatide group, 46 (94%) also received continuous infusion of insulin due to insufficient glycemic control with exenatide alone.

**Table 1:** Baseline characteristics of patients in the control and exenatide groups

	Control group (n = 43)	Exenatide group (n = 49)	Standardized difference
Age (years)	69 [61–76]	71 [63–75]	− 0.26
Males^a^	36 (84%)	46 (94%)	− 0.33
ASA physical status III^a^	41 (95%)	46 (94%)	0.06
Comorbities^a^			
Smoking	21 (49%)	28 (57%)	− 0.17
Hypertension	30 (70%)	29 (59%)	0.22
Dyslipidemia	25 (58%)	30 (61%)	− 0.06
Obesity (BMI ≥ 30 kg m^−2^)	10 (23%)	7 (14%)	0.23
Diabetes mellitus	9 (21%)	10 (20%)	0.01
Creatinine clearance (ml min^−1^)	82 [74–93]	82 [75–90]	0.02
Fasting blood glucose (mg dl^−1^)	105 [99–115]	101 [94–112]	0.23
Glycated hemoglobin (%)	5.7 [5.5–6.1]	6.0 [5.6–6.3]	− 0.33
Euroscore	4.8 [3.5–7.3]	5.4 [4.6–7.3]	− 0.07
Left ventricular ejection fraction (%)^a^			
< 30	0 (0%)	1 (2%)	
30–50	6 (14%)	15 (31%)	
> 50	37 (86%)	33 (67%)	
Surgical procedure			
Number of bypass grafts	3 [2–4]	3 [2–4]	− 0.30
Duration of surgery (min)^b^	237 (58)	251 (58)	− 0.24
On-pump surgery	29 (67%)	33 (67%)	0.00
Duration of ECC (min)^a^	67 [53–84]	71 [59–91]	− 0.23
Duration of aortic cross clamping (min)^a^	61 [46–89]	63 [52–79]	0.16
Maintenance of anesthesia			
Sevoflurane^a^	8 (19)	11 (22)	0.80
Propofol^a^	14 (32)	16 (33)	1.00
Propofol and sevoflurane^a,c^	21 (49)	22 (45)	0.83

Data are median [interquartile range 25–75%]

*ASA* American Society of Anesthesiologists, *BMI* body mass index, *ECC* extracorporeal circulation

^a^Data are number of patients (percentage)

^b^Data are mean (standard deviation)

^c^General anesthesia was maintained with inhalation of sevoflurane until the start of cardiopulmonary bypass; then, the inhalation of sevoflurane was definitely stopped and target-controlled infusion of propofol was started and was proceeded until the end of general anesthesia

### Troponin and BNP values

**Fig. 2:**
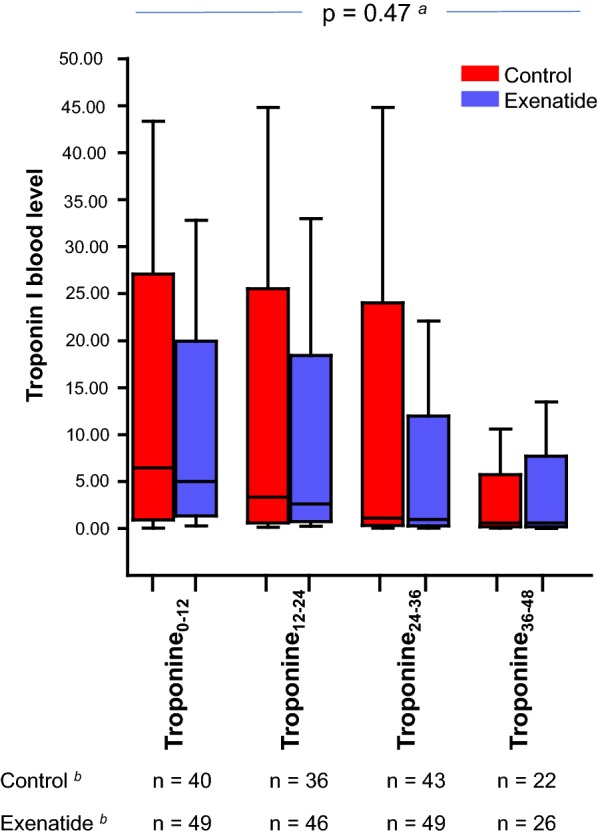
Troponin values in the cardiac surgery intensive care unit. Troponin_0–12_, troponin_12–24_, troponin_24–48_, and troponin_48–72_ are the highest troponin values measured respectively within 0–12 h, 12–24 h, 24–48 h, and 48–72 h after admission to the cardiac surgery intensive care unit. ^a^p-value for repeated measures ANOVA. ^b^Number of troponin measurements for each timepoints in the control and in the exenatide groups

Plasma concentration of troponin_12–24_ did not significantly differ between the control (3.34 [1.06–6.19] µg l^−1^) and the exenatide (2.64 [1.29–3.85] µg l^−1^) groups (mean difference [95% confidence interval] 0.16 [− 0.25; 0.57], p = 0.54) (Fig. 2). The troponin values at the different measurement timepoints are presented in Fig. 2. The peak post-operative troponin (6.34 [1.36–10.90] versus 5.04 [2.39–7.18] µg l^−1^, mean difference [95% confidence interval] 0.20 [− 0.22; 0.61], p = 0.39; respectively in the control and exenatide groups) and plasma concentration of troponin I during the first 72 h in the Cardiac Surgery ICU were not significant different between groups (p = 0.47, Fig. 2). The AUC_troponin_ did not significantly differ between groups (108 [53–258] versus 113 [58–173], mean difference [95% confidence interval] 0.03 [− 0.36; 0.43], p = 0.71, respectively in the control and exenatide groups). The median number of troponin assays performed during the ICU stay was 5 [4–6] versus 5 [4, 5] in the control versus exenatide groups (p = 0.55).

**Fig. 3:**
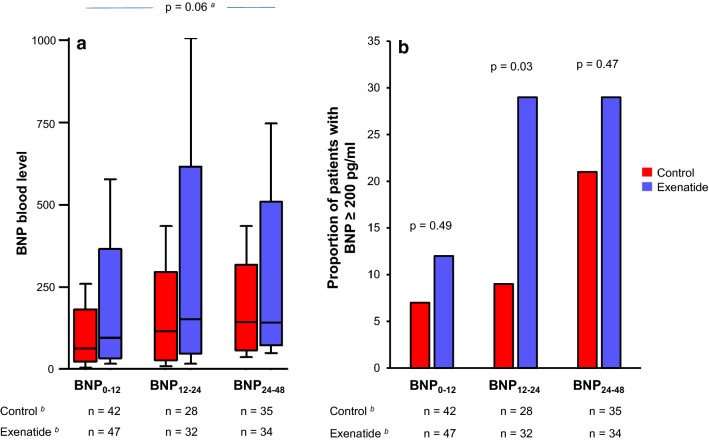
Brain natriuretic peptide (BNP) values (**a**) and proportion of patients with BNP ≥ 200 pg/ml (**b**) in the cardiac surgery intensive care unit. BNP_0–12_, BNP_12–24_ and BNP_24–48_ are the highest BNP values measured within respectively 0–12 h, 12–24 h and 24–48 h after admission to the Cardiac Surgery Intensive Care Unit. ^a^p-value for repeated measures ANOVA. ^b^Number of BNP measurements for each timepoints in the control and in the exenatide groups

Plasma concentrations of BNP during the first 48 h in the ICU after surgery did not significantly differ between the control and exenatide groups (p = 0.06, Fig. 3). The details of BNP levels and the proportion of patients with BNP_0–12_, BNP_12–24_ or BNP_24–48_ ≥ 200 pg ml^−1^ are given in Fig. 3.

### Left ventricular function

No patient in the control group and only 2 patients (4%) in the exenatide group received dobutamine during the ICU stay (relative risk [95% confidence interval] 1.04 [0.98; 1.10], p = 0.50).

The number of patients with LVEF < 50% at the follow-up consultation was 5 (12%) versus 8 (16%) (relative risk [95% confidence interval] 0.68 [0.16; 2.59], p = 0.56) in the control and exenatide groups, respectively. No patient had LVEF < 30% at follow-up.

### Quality of life

The values of the dimensions of the SF36 questionnaire at inclusion, 1, 3, 6 and 12 months were not significantly different between the control and exenatide groups (data no shown).

## Discussion

This sub study of a randomized clinical phase II/III trial suggests that iv exenatide for perioperative glycemic control after scheduled CABG surgery does not procure any cardioprotective effect and does not appear to improve cardiac function in the short or medium term.

The cardioprotective effect of GLP-1 receptor agonists or GLP-1 metabolites has been largely explored and reported in animal models of ischemia-reperfusion injury [4–10, 29]. GLP-1 analogs administration during ischemia and/or reperfusion inhibits the process that leads to apoptosis of myocardial cells during the reperfusion phase [4, 6]. The effects of GLP-1 and its analogs are mediated via the G-protein-coupled GLP-1 receptor, leading to activation of intracellular signaling pathways involved in ischemic pre- and post-conditioning, notably the Reperfusion Injury Salvage Kinase (*RISK*) pathway [4, 6, 19], and opening of mitochondrial K^+^-ATP channels [30]. In a randomized study, Lonborg et al. [15, 16] reported a cardioprotective effect of intravenous exenatide in patients with ST-segment elevation myocardial infarction. There are several possible explanations for the discrepancies observed between our results and previous studies.

Firstly, the modalities of intravenous administration of exenatide in our study were fixed by monitoring of blood glucose, with treatment initiated as soon as the first blood glucose value exceeded ≥ 140 mg dl^−1^, i.e. within an average time of 3 h after admission to the Cardiac Surgery ICU [20]. Most studies addressing the cardioprotective effects of other drugs suggested that the greatest improvement in myocardial ischemia-reperfusion injuries could be obtained when administered during the early phase of reperfusion. In the study by Lonborg et al. [15, 16], exenatide was initiated during the ischemia phase and continued for 6 h after coronary artery reperfusion. The authors reported a reduction in final infarct size among patients who received exenatide [15, 16]. In a similar population, intravenous exenatide was associated with a reduction in plasma levels of troponin I and CK-MB during the first 72 h, and an improvement in subclinical left ventricular function at 6 months [17]. In contrast, a recent randomized, placebo-controlled trial by Wiberg et al. [31] failed to find any neuroprotective effects of delayed exenatide administration during the reperfusion phase of out-of-hospital cardiac, even though the mechanisms mediating the cardioprotective and neuroprotective effects of exenatide are similar. Thus, the delay from the start of reperfusion to the initiation of exenatide might have been too long to provide a significant cardioprotective effect. Accordingly, a reduction in the extent of ischemia-reperfusion lesions thanks to exenatide could be obtained on condition that the treatment is initiated during the ischemia phase.

Beyond the delay from the start of reperfusion, the beneficial effects of GLP-1 administration during reperfusion could be independent of the GLP-1 receptor pathway, and involve the GLP-1(9–36) amide, which results from the breakdown of GLP-1 by the plasma enzyme dipeptidyl peptidase-IV [3, 10]. This hypothesis could explain the lack of cardioprotective effect of exenatide administered during the reperfusion phase.

Secondly, the dose of exenatide prescribed in our study could be insufficient to generate a significant cardioprotective effect. The dosing regimen used in this study was mandated by the national agency for the safety of health products (Agence Nationale de Sécurité du Médicaments et des produits de santé, ANSM), because the use of intravenous exenatide in our study was off-label. Therefore, the dose regimen imposed was that used in the only study of exenatide for the control of stress hyperglycemia available at the time of the design of our study [32]. While it is difficult to compare equivalence between GLP-1 and exenatide, or between human and animal studies, it nonetheless seems that the dose of exenatide administered plays a key role in mediating the protective effects [17]. In this regard, Bernik et al. failed to observed any protective effect with a lower dose of exenatide in the context of acute myocardial infarction [33], despite similar timing of administration to the studies by Lonborg et al. [15, 16].

Thirdly, the body of literature reporting protective effects with GLP-1 analogues comprised mostly studies comparing the study drug to a control group treated by placebo. In our study, all patients in the control group received insulin. A reduction in ischemia-reperfusion injury has been described with the use of insulin [34–37], which could explain why no benefit of exenatide was observed as compared to the control group. Moreover, inflammation-induced increase of GLP-1 has been reported after cardiac surgery [38] and could have lowered the intergroup differences. The lack of any additional cardioprotective effect of iv exenatide compared to iv insulin does not mean that exenatide, and other GLP-1 agonists, has no cardioprotective effect in cardiac surgery patients. Insulin and other medications, such a sevoflurane, could have activated and then saturated all of the cardioprotective pathways, that were desensitized to any further effect of exenatide [37, 39, 40]. In particular, insulin and exenatide appeared to exert most of their cardioprotective effects by increasing myocardial glucose uptake [39]. Combining the GLP-1 agonist liraglutide and insulin did not appear to provide any additional effect compared to liraglutide or insulin alone in type 2 diabetes mellitus [41]. Moreover, a low overall baseline insulin resistance in non-diabetic patients in one hand, and desensitized cardioprotective pathways related to altered sarcolemma function of cardiomyocytes in long-standing diabetes mellitus in the other hand, could contribute to an impaired response to exenatide in the present study [42, 43]. Furthermore, early and prolonged exenatide administration, and the use of a long-acting GLP-1 agonist, such as liraglutide, could provide a higher cardioprotective effect, as reported by Chen et al. [44] in myocardial infarction. Finally, by treating hyperglycemia and by favoring hypoglycemia, both exenatide and insulin could have lowered the activation of the cardioprotective pathways [43, 45].

Inotropic effects of GLP-1 that could improve quality of life and functional symptoms has been reported in patients with chronic heart failure [14], in patients with myocardial infarction and left ventricular dysfunction [13], and in CABG patients [11, 12]. In the present study, exenatide failed to improve either left ventricular ejection fraction, use of inotropic agents after scheduled CABG surgery. These results are consistent with recently published data [46, 47]. Indeed, in a recent meta-analysis, liraglutide but not exenatide appeared to share the inotropic effects of GLP-1 [46]. In fact, as shown by the low rate of use of dobutamine and the low levels of plasma BNP observed during the first 48 h in the ICU, only few patients had significant heart failure after surgery. However, it would appear that the inotropic effect of GLP-1 is only exerted in the context of severe heart failure [8, 9, 48]. The absence of an impact of exenatide on quality of life after CABG surgery remains difficult to interpret, in view of the small sample size, and the multifactorial nature of quality of life perception.

### Study limitations

This study suffers from some limitations that deserve to be underlined. Firstly, this is a sub study of a phase II/III randomized trial, and thus, the mode of administration of exenatide was not originally designed for the purposes of evaluating the potential cardioprotective effect of exenatide. Furthermore, the inclusion criteria of the main study selected a population of patients at low risk of post-operative heart failure. We therefore cannot rule out the possibility that different results might be observed if the dose regimen of exenatide or if the patient profiles were different. Secondly, no proper sample size calculation could be performed prior to the analysis in the absence of data in cardiac surgery patients, and the study suffered from a lack of power. Based on our results, 800 patients in each group would be required to reach a statistically significant difference in the primary outcome.

## Conclusions

This sub study of a randomized, phase II/III trial failed to observe any additional cardioprotective effect of iv exenatide compared to iv insulin following CABG surgery in a population of patients at low risk of post-operative heart failure. Both iv insulin and iv exenatide might have activated cardioprotective pathways in a similar extent. The discrepancy between our findings and previous animal and clinical studies warrant further exploration in studies designed specifically to evaluate the potential protective effects of exenatide, or of other GLP-1 agonists, such as liraglutide, in the context of cardiac surgery.

## Abbreviations

CABG: coronary artery bypass graft
GLP-1: glucagon-like peptide 1
IV: intravenous
ASA: American Society of Anesthesiologists
ICU: intensive care unit
BNP: brain natriuretic peptide
LVEF: left ventricular ejection fraction
